# Fatal poisonings in Oslo: a one-year observational study

**DOI:** 10.1186/1471-227X-10-13

**Published:** 2010-06-06

**Authors:** Mari A Bjornaas, Brita Teige, Knut E Hovda, Oivind Ekeberg, Fridtjof Heyerdahl, Dag Jacobsen

**Affiliations:** 1 Department of Acute Medicine, Oslo University Hospital Ulleval, Kirkeveien 166, N-0407 Oslo, Norway; 2 Institute of Forensic Medicine, University of Oslo, Gaustadalléen 30, N-0027 Oslo, Norway

## Abstract

**Background:**

Acute poisonings are common and are treated at different levels of the health care system. Since most fatal poisonings occur outside hospital, these must be included when studying characteristics of such deaths. The pattern of toxic agents differs between fatal and non-fatal poisonings. By including all poisoning episodes, cause-fatality rates can be calculated.

**Methods:**

Fatal and non-fatal acute poisonings in subjects aged ≥16 years in Oslo (428 198 inhabitants) were included consecutively in an observational multi-centre study including the ambulance services, the Oslo Emergency Ward (outpatient clinic), and hospitals, as well as medico-legal autopsies from 1st April 2003 to 31st March 2004. Characteristics of fatal poisonings were examined, and a comparison of toxic agents was made between fatal and non-fatal acute poisoning.

**Results:**

In Oslo, during the one-year period studied, 103 subjects aged ≥16 years died of acute poisoning. The annual mortality rate was 24 per 100 000. The male-female ratio was 2:1, and the mean age was 44 years (range 19-86 years). In 92 cases (89%), death occurred outside hospital. The main toxic agents were opiates or opioids (65% of cases), followed by ethanol (9%), tricyclic anti-depressants (TCAs) (4%), benzodiazepines (4%), and zopiclone (4%). Seventy-one (69%) were evaluated as accidental deaths and 32 (31%) as suicides. In 70% of all cases, and in 34% of suicides, the deceased was classified as drug or alcohol dependent. When compared with the 2981 non-fatal acute poisonings registered during the study period, the case fatality rate was 3% (95% C.I., 0.03-0.04). Methanol, TCAs, and antihistamines had the highest case fatality rates; 33% (95% C.I., 0.008-0.91), 14% (95% C.I., 0.04-0.33), and 10% (95% C.I., 0.02-0.27), respectively.

**Conclusions:**

Three per cent of all acute poisonings were fatal, and nine out of ten deaths by acute poisonings occurred outside hospital. Two-thirds were evaluated as accidental deaths. Although case fatality rates were highest for methanol, TCAs, and antihistamines, most deaths were caused by opiates or opioids.

## Background

Deaths by acute poisoning are mainly suicides or consequences of substance use disorders. The majority of deaths attributed to substance use disorder are considered accidental, i.e. death was not the intended outcome [1]. However, a post-mortem determination of the intention behind a fatal intake is uncertain. Some suicides might be classified as accidental deaths, and vice versa [2]. Furthermore, self-destructiveness is a common feature among those with suicidal behaviour and among those repeatedly treated for accidental overdoses [3]. This may explain why the evaluated intention in repeated acute poisonings often changes from one admission to another [4]. Hence, the inclusion of all deaths by acute poisoning will give a more complete picture of mortality and toxic agents used among this group of people with unnatural deaths.

The changing availability of drugs influences the pattern of toxic agents in fatal poisonings [5-7]. During recent decades there has been a shift in prescriptions from tricyclic anti-depressants (TCAs) to newer selective serotonin reuptake inhibitors (SSRIs) and other anti-depressants, although the recent controversy regarding suicide risk is still debated [8,9]. The implementation of methadone maintenance treatment has led to an increase in deaths related to methadone intake [7], but the magnitude of the increase varies between countries [1]. Regular updates on the pattern of toxic agents used are therefore of interest, as it is important in the discussion of prescription policy and treatment of drug addiction. Death certificates seldom include additional agents according to the Anatomical Therapeutic Chemical (ATC) classification system [10], and the coding of ethanol poisoning is problematic in the International Classification of Diseases (ICD) system. Important information regarding toxic patterns is therefore lost if studies are based solely on death certificates and mortality statistics [10,11]. Studies designed to examine the patterns of both main and additional agents in acute poisonings are therefore necessary.

In order to describe the pattern of poisoning it would be useful to compare the toxic agents used in fatal versus non-fatal poisonings, and hence the relative influence of each agent on mortality rates. Case fatality rates can be calculated as long as all fatal poisonings in a defined area are known, along with the number of diagnosed non-fatal acute poisonings.

The aim of the present study was to describe the pattern of drugs detected in fatal acute poisonings in Oslo during one year, including deaths both in and outside hospitals.

## Methods

Acute poisonings in subjects aged 16 years or older occurring in Oslo were included consecutively in an observational multicentre study from 1st April 2003 to 31st March 2004. This was a prospective study using a standardized data collection form, and the cases were included consecutively. The complete study involved the four hospitals in Oslo that treat patients with acute poisoning, the Oslo Emergency Ward (outpatient clinic), the ambulance service in Oslo, and the Institute of Forensic Medicine, University of Oslo. The patients were included on admission. All contacts were recorded, and each poisoning episode was traced through different levels of care using of each patient's unique personal identification number. This also included those found dead and subjected to medico-legal examination. Thus, a complete one-year picture of all patients in the capital of Norway who were in contact with health care services because of acute poisoning was obtained. The catchment area had a total population of 521 886, of which 428 198 were older than 16 years (1st January 2004). Data on fatal poisoning is presented here. The intention behind the fatal intake, the history of substance use disorder, and previous suicide attempts were assessed. Furthermore, the main toxic agents in fatal and non-fatal acute poisonings in the same geographical area and period were compared, and case-fatality rates were calculated. Epidemiological data on the non-fatal poisonings has been presented previously [12,13].

### Data collection

The inclusion criterion for the present part of the study was exposure to a toxic substance in an amount leading to death in subjects ≥16 years, i.e. a primary diagnosis of acute poisoning. Deaths both in and outside hospitals were included. Furthermore, both deliberate acute poisonings and accidental poisonings were included. Exclusion criteria were chronic poisoning and admission to hospital or the Institute of Forensic Medicine with another *primary *diagnosis, such as trauma, even if intoxication (mostly from ethanol) was the underlying reason for the accident.

Medico-legal autopsies were performed at the Institute of Forensic Medicine, University of Oslo. The Institute of Forensic Medicine examines all deaths due to poisoning, according to Norwegian law. Forensic pathologists at the Institute perform the autopsies. Police records are available if relevant. In all forensic cases, toxicological analyses were performed at the Norwegian Institute of Public Health, Division of Forensic Toxicology and Drug Abuse.

For deaths occurring in hospitals where a medico-legal autopsy was not performed, physicians obtained data by completing a standardized registration form based on medical files. If the patient had been conscious during the hospital stay, the form was completed based on a clinical interview.

### Classification of toxic agents

Toxic agents were recorded and classified as main or additional agents. Toxicologists at the Institute of Forensic Toxicology and the forensic pathologist did the determination of the toxic agents responsible for the death in the fatal cases. However, for patients who survived, and for fatal poisonings in hospital not subjected to medico-legal autopsy, the treating physician made the determination of the toxic agents leading to hospitalization.

For all medico-legal autopsies, drug screening was performed. The main toxic agent in fatal poisonings was defined as the substance supposed to be the main contributor to death. Toxicological analyses included a drug-screening program. According to the Institute of Forensic Toxicology's standard protocol, blood from the common iliac vein was used. Alcohols (ethanol, methanol, isoproanol and aceton) were analysed with headspace gas chromatography (GC), and two different columns were used. Immunological screening was used for amphetamines, cannabis (tetrahydrocannabinol, THC), cocaine/benzoylecognin, opiates and opioids, and positive results were confirmed using GC-mass spectrometry (GC-MS). Liquid chromatography single stage mass spectrometry (LC-single MS) was used for benzodiazepines and their metabolites, and for 63 of the most commonly encountered drugs within the given groups: analgesics, anti-depressants, neuroleptics, anti-epileptics, and others. Confirmation tests used also LC-single MS, but with different extraction and separation columns. In addition, carbon monoxide was analysed for. Other drugs were analysed on request.

Heroin metabolizes quickly to morphine via 6-monoacetylmorphine (6-MAM). If 6-MAM is not detected in blood or urine, it is not possible to tell from the analytical results if heroin or morphine was the initial compound. Therefore, these deaths were registered as heroin/morphine deaths.

The main toxicological agent in fatal poisonings was determined by the forensic pathologist.

For patients who survived, and for fatal poisonings in hospital not subjected to medico-legal autopsy, the main toxic agent was defined as the substance supposed to be most toxic considering the amount taken. Other agents were defined as additional agents. The evaluation was made by the treating physician based on all available information. A drug screen was not routinely performed but was conducted if requested by the physician (e.g. ethanol and paracetamol in most cases). Information obtained at the highest level of care was chosen if the patient was treated at different health care levels, i.e. if the patient was treated both by ambulance services and in hospital, data from the hospital was used in further analyses. For hospitalized patients, the mean blood alcohol concentration was 1.77 % (range 0.2 - 6.2) if ethanol was identified, and if it was found to be the main toxic agent, the mean blood concentration was 2.26 % (range 0.2 - 6.2). For the ambulance service, blood concentration levels were not available. In the Oslo Emergency Ward, blood concentration levels were available to the physicians evaluating the main and additional agents, but these figures were not available to the researchers. For fatal poisonings, the alcohol concentration levels were not available to the researchers, but they were available to the forensic pathologist classifying the toxic agents for each patient.

### Evaluated intention and substance abuse

The intention behind the fatal poisoning was classified as suicide or accidental death. Evaluation of intention was based on all information available in each case, including patients' own reported intentions, when known. Special attention was given to letters confirming suicidal intent, supposed intake of lethal doses of the toxic agent(s), or other active procedures to ensure a lethal outcome. Information from other sources such as ambulance personnel and companions was also taken into consideration. In the forensic cases, the evaluation of intention was according to the assessment of the forensic pathologist. In fatal poisonings not subjected to medico-legal autopsy, the attending physician classified the intention.

Substance use disorders were classified according to the ICD-10 criteria [14], i.e. drug dependence as for ethanol, prescription drugs, or illegal drugs. One category was chosen in each case, but among those who were dependent on illegal drugs, six patients fulfilled the criteria for other substance use disorders as well: four as ethanol dependent, and two as dependent on prescription drugs.

### Statistics

The standardized registration forms were optically scanned and processed using TeleForm Desktop version 9.1 (TeleForm, Verity Inc., Sunnyvale, California). Statistics were analysed using SPSS, version 16.0 (SPSS, Chicago, Illinois), except 95% confidence intervals for case fatality rates, where NCSS version 2007 (NCSS, Kaysville, Utah) was used. An independent samples *t *test was used to compare continuous data, and χ^2 ^tests were used to compare categorical data.

### Ethics

The study was carried out according to the Helsinki declaration. Permission was obtained from the National Data Inspectorate and the Regional Ethics Committee. The links between patients' names and social security numbers and the study case numbers were stored by Statistics Norway.

## Results

**Figure 1:**
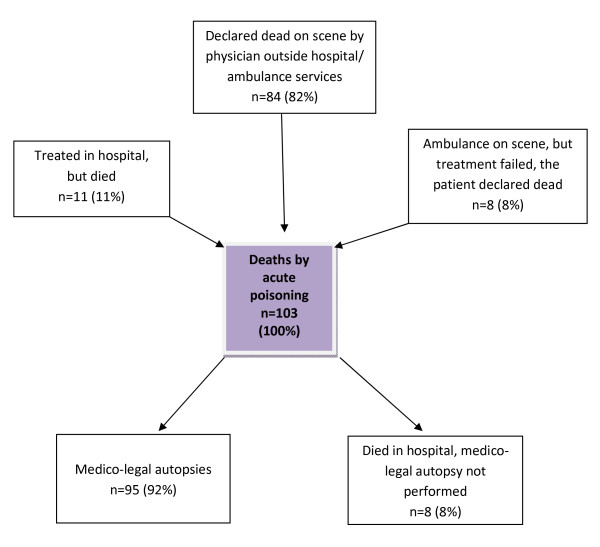
**Deaths by acute poisoning in Oslo during one year**.

During one year, 103 subjects aged 16 years or older died of acute poisoning in Oslo, giving an annual mortality rate of 24 per 100 000 for Oslo. Eleven subjects (11%) were treated in hospital because of acute poisoning but died in spite of treatment (Figure 1), of whom three were medico-legally examined. In one of these cases, the death was not registered as caused by acute poisoning at the time of death. Eight people (8%) treated on scene by ambulance services were declared dead on scene, whereas 84 (82%) were declared dead on scene by physicians outside hospital or ambulance services.

Sixty-nine (67%) of all deaths were males (Table 1). The mean age was 44 years (range 19-86 years); 42 years among males and 49 years among females (p = 0.025). Ninety-three (90%) were originally from Norway. In eight cases, the deceased had previously been treated by ambulance services because of acute poisoning in the same year. Two other people had been treated in hospital during the same year, and one person had been treated both by ambulance services and in hospital on different occasions during the study period.

**Table 1:** Characteristics of fatal poisonings in Oslo during one year.

	Males n = 69 (%)	Females n = 34 (%)	Total n = 103 (%)
Mean age	42	49	44

Country of origin			
Norway	61 (88)	32 (94)	93 (90)
Other	5 (7)	3 (4)	6 (6)
Unknown	1 (3)	1 (3)	4 (4)

Suicide letter	3 (4)	1(3)	4 (4)

Previous suicide attempt during lifetime	9 (13)	6 (18)	15 (15)

Drug dependence			
None	4 (6)	5 (15)	9 (9)
Ethanol	9 (13)	3 (9)	12 (12)
Prescription drugs	1 (1)	5 (15)	6 (6)
Illegal drugs	42 (61)	12 (35)	54 (52)
Unknown	13 (19)	9 (26)	22 (21)

Evaluated intention			
Suicide	15 (22)	17 (50)	32 (31)
Accidental death	54 (78)	17 (50)	71 (69)

The place of death was at home (n = 53, 51%), other private locations (n = 15, 15%), in hospitals (n = 11, 11%), outdoors (n = 9, 9%), other institutions (n = 2, 2%), public restroom (n = 1, 1%) and unknown (n = 13, 13%). Two were psychiatric in-patients at the time of death; one of these cases was evaluated as suicide, one as accidental death.

### Pattern of main toxic agents

Opiates or opioids were the most frequent main toxic agents, accounting for 68 (66%) deaths (Table 2). Fifty-two (50% of total poisonings) were heroin- or morphine-related deaths, six (6%) were related to methadone, five (5%) to codeine, and in four (4%), the specific compound was unknown. Ethanol was the second most common main toxic agent (n = 9, 9%). TCAs, benzodiazepines, and zopiclone accounted for four (4%) fatal poisonings each. Paracetamol was the main toxic agent in two (2%) of the cases. In forty-five (44%) cases the drug had presumably been taken orally, 47 (46%) subjects had injected the drug, four (4%) had inhaled the drug, and in seven (7%) cases, the method of administration was unknown.

**Table 2:** Main and additional toxic agents in fatal poisonings in Oslo during one year.

	Main agents	Additional agents*	Sum*
	n = 103 (%)	n = 103 (%)	n = 103 (%)
**Opiates/opioids**	**68 (66)**	**10 (10)**	**78 (76)**
Morphine/heroin	52 (50)	-	52 (50)
Methadone	6 (6)	5 (5)	11 (11)
Codeine	5 (5)	-	5 (5)
Dextropropoxyphene	1 (1)	3 (3)	4 (4)
Unknown	4 (4)	2 (2)	6 (6)

**Ethanol**	**9 (9)**	**18 (17)**	**27 (26)**

**Tricyclic antidepressants**	**4 (4)**	**5 (5)**	**9 (9)**

**Benzodiazepines**	**4 (4)**	**74 (72)**	**78 (76)**
Flunitrazepam	1 (1)	15 (15)	16 (16)
Diazepam	3 (3)	35 (34)	37 (36)
Other	-	24 (23)	24 (23)

**Zopiclone**	**4 (4)**	**7 (7)**	**11 (11)**

**Antihistamines**	**3 (3)**	**4 (4)**	**7 (7)**

**Paracetamol**	**2 (2)**	**11 (11)**	**13 (13)**

**Carbon monoxide**	**2 (2)**	**4 (4)**	**6 (6)**

**Neuroleptics**	**2 (2)**	**15 (15)**	**17 (17)**

**Cocaine**	**1 (1)**	**2 (2)**	**3 (3)**

**Selective serotonin reuptake inhibitors**	**1 (1)**	**14 (14)**	**15 (15)**

**Anti-epileptics**	**1 (1)**	-	**1 (1)**

**Methanol**	**1 (1)**	-	**1 (1)**

**Carisoprodol**	**1 (1)**	**6 (6)**	**7 (7)**

**Amphetamines**	-	**16 (16)**	**16 (16)**

**Cannabis/THC**	-	**13 (13)**	**13 (13)**

**Other anti-depressants**	-	**8 (8)**	**8 (8)**

**Other sedatives**	-	**2 (2)**	**2 (2)**

**Salicylic acid**	-	**1 (1)**	**1 (1)**

**Total**	**103 (100)**	**210**	**313**

*More than one additional agent per patient was registered if appropriate.

### Additional agents

Benzodiazepines were the most common additional agents, found in 74 (72%) of the cases (Table 2). Ethanol was second most common, with 18 cases (17%), followed by amphetamines (16%), neuroleptics (15%), SSRIs (14%), cannabis or tetrahydrocannabinol (THC) (13%), paracetamol (11%), opiates or opioids (10%), other anti-depressants (8%), and TCAs (5%).

### Intention

Thirty-two (31%) of the deaths were suicides, and 71 (69%) were accidental deaths (Table 1). Among females, 17 (50%) of deaths were evaluated as suicides, compared with 15 (22%) among males (p = 0.010). Written suicide letters were found in four (4%) of the cases, all were evaluated as certain suicide. There was information regarding previous suicide attempts in 15 (15%) of the cases: nine (13%) among males and six (17%) among females.

### Substance use disorders

Seventy per cent of the deceased were diagnosed post-mortem with a substance use disorder (Table 3). Fifty-four (52%) were evaluated as illegal drug dependent: 42 (60%) of males and 12 (35%) of females. Ethanol dependency was found among 12 (12%): nine (23%) of males and three (35%) of females. Six people (6%) were dependent on prescription drugs. Among those evaluated as suicides, three were classified as illegal drug dependent (9% of all suicides) (Table 3). Four were ethanol dependent (13%), and four were dependent on prescription drugs (13%). One-third (34%) of those who committed suicide had substance use disorders.

**Table 3:** Evaluated intention in fatal poisoning and history of substance use disorders prior to death.

Substance use disorder	Suicide n (%)	Accidental death n (%)	Total n (%)
**None**	**8 (25)**	**1 (1)**	**9 (9)**

**Drug dependence**	**11 (34)**	**61 (86)**	**72 (70)**

Ethanol	4 (13)	8 (11)	12 (12)

Prescription drugs	4 (13)	2 (3)	6 (6)

Illegal drugs	3 (9)	51 (72)	54 (52)

**Unknown**	**13 (41)**	**9 (13)**	**22 (21)**

**Total**	**32 (101)**	**71 (100)**	**103 (100)**

### Toxic agents according to intention and substance use disorder

Opiates or opioids were the most commonly found main toxic agent in both suicides and accidental deaths, found in 44% and 76% of the cases, respectively (Table 4). In the remaining cases of suicide, a spectrum of toxic agents was used. Ethanol was the second most common main toxic agent in accidental deaths (11%).

**Table 4:** Evaluated intention and main toxic agents in fatal poisonings in Oslo during one year.

Main toxic agents	Suicide n (%)	Accidental death n (%)	Total n (%)
**Opiates/opioids**	14 (44)	54 (76)	68 (66)

**Ethanol**	1 (3)	8 (11)	9 (9)

**Benzodiazepines**	3 (9)	1 (1)	4 (4)

**Paracetamol**	2 (6)	-	2 (2)

**Methanol**	-	1 (1)	1 (1)

**Neuroleptics**	2 (6)	-	2 (2)

**Cocaine**	-	1 (1)	1 (1)

**Tricyclic antidepressants**	2 (6)	2 (3)	4 (4)

**Selective serotonin reuptake inhibitors**	-	1 (1)	1 (1)

**Antihistamines**	2 (6)	1 (1)	3 (3)

**Zopiclone**	2 (6)	2 (3)	4 (4)

**Carisoprodol**	1 (3)	-	1 (1)

**Anti-epileptics**	1 (3)	-	1 (1)

**Carbon monoxide**	2 (6)	-	2 (2)

**Total**	**32 (98)**	**71(98)**	**103 (101)**

Anti-depressants (SSRIs, TCAs, or others) were found as the main or additional toxic agent in 28 (27%) of the deaths: 15 (22%) of men, and 13 (38%) of women. Thirty-one per cent of those who committed suicide had taken anti-depressants (n = 10), as had 25% of those with accidental death (n = 18). Among those who were alcohol dependent, 42% (n = 5) had taken anti-depressants. Seventeen per cent of illegal drug abusers had used anti-depressants (n = 9), whereas 50% of those abusing prescription drugs had used such agents (n = 3).

### Toxic agents in fatal vs. non-fatal acute poisonings

During the study period, 2998 acute poisoning episodes were registered in Oslo. Of these, 103 were fatal, of whom one episode were recognized as result of fatal poisoning only at the autopsy, and therefore not included in studies presented earlier [4,12]. The percentage of deaths per acute poisoning episode was 3% in total (95% C.I., 0.03-0.04). Opiates or opioids were the main toxic agent in the majority of the fatal poisonings, whereas ethanol, opiates or opioids, and benzodiazepines dominated among those who survived the acute poisoning episode (Table 5). The case fatality rate was 0.07 or 7% for opiates or opioids (95% C.I., 0.06-0.09) and 0.9% for ethanol (95% C.I., 0.004-0.02). Acute poisonings caused by methanol, TCAs, and antihistamines resulted most often in fatal poisonings when the total number of such poisonings was taken into account. Methanol poisoning was fatal in 33% of the cases (95% C.I., 0.008-0.91), TCAs in 14% (95% C.I., 0.04-0.33), and antihistamines in 10% (95% C.I., 0.02-0.27) of all registered poisoning episodes by these substances, respectively.

**Table 5:** Comparison of main toxic agents between fatal and non-fatal acute poisonings during one year in Oslo.

		Main agents in acute poisonings		Case fatality rates	
		**Fatal**	**Non-fatal**	**Fatal cases/all cases**	
		**n (%)**	**n (%)**		**95% C.I**.

**>5% fatality rate**	Methanol	1 (1)	2 (0.1)	1/3 = 0.33	0.008-0.91
	TCAs**	4 (4)	24 (0.8)	4/28 = 0.14	0.04-0.33
	Antihistamines	3 (3)	27 (0.9)	3/30 = 0.10	0.02-0.27
	Zopiclone	4 (4)	45 (2)	4/49 = 0.08	0.02-0.20
	Opiates/opioids	68 (66)	847(28)	68/915 = 0.07	0.06-0.09
	Carbon monoxide	2 (2)	31 (1)	2/33 = 0.06	0.007-0.20

**<5% fatality rate**	Cocaine	1 (1)	23 (1)	1/24 = 0.04	0.001-0.21
	Anti-epileptics	1 (1)	33 (1)	1/34 = 0.03	0.0007-0.15
	SSRIs**	1 (1)	45 (2)	1/46 = 0.02	0.0005-0.11
	Neuroleptics	2 (2)	79 (3)	2/81 = 0.02	0.003-0.09
	Carisoprodol	1 (1)	57 (2)	1/58 = 0.02	0.0004-0.09
	Paracetamol	2 (2)	142 (5)	2/144 = 0.01	0.002-0.05
	Benzodiazepines	4 (4)	261 (9)	4/265 = 0.01	0.004-0.04

**<1% fatality rate**	Ethanol	9 (9)	984 (33)	9/993 = 0.009	0.004-0.02

**No fatalities**	Cannabis/THC	-	2 (0.1)	0/2 = 0	0-0.84
	Salicylate	-	3 (0.1)	0/3 = 0	0-0.71
	Ethylene glycol	-	4 (0.1)	0/4 = 0	0-0.60
	Lithium	-	6 (0.2)	0/6 = 0	0-0.46
	Ecstasy	-	9 (0.3))	0/9 = 0	0-0.34
	Other sedatives	-	14 (0.5)	0/14 = 0	0-0.23
	Cardiovascular agents	-	15 (0.5)	0/15 = 0	0-0.22
	NSAIDs**	-	16 (0.5)	0/16 = 0	0-0.21
	Amphetamine	-	53 (2)	0/53 = 0	0-0.07
	GHB**	-	97 (3)	0/97 = 0	0-0.04
	Others	-	78 (3)	-	-
	Unknown*	-	63 (2)	-	-

	**Total**	**103 (100)**	**2981 (100)**	**103/2998 = 0.03**	**0.03-0.04**

The toxic agents are reported according to their case fatality rate.

*Cases treated by the ambulance services as acute poisonings, but where the toxic agent was unknown or not registered.

**TCAs: tricyclic antidepressants, SSRIs: selective serotonin reuptake inhibitors, GHB: gamma-hydroxybutyrate, NSAIDs: non-steroid anti-inflammatory drugs

## Discussion

Three per cent of all acute poisonings were fatal. Previous studies of acute poisonings have focused on in-hospital mortality [15,16], although a register-based study for the whole of Norway found that 80% of deaths occurred outside hospital [11]. In the present study from Oslo, the mortality rate was 24 per 100 000 inhabitants, whereas the register-based study including all of Norway found a lower mortality rate (10.8 per 100 000) [11]. This may indicate a higher mortality rate in Oslo, an urban setting, which usually has a larger population of drug addicts compared with the average of the whole country, including rural areas. However, it may also indicate that retrospective register-based studies tend to underestimate mortality.

In the present study, the in-hospital mortality of 1.1% was comparable to other studies [17]. However, focusing solely on in-hospital mortality tends to underestimate mortality rates, and in the present study, the relative importance of drugs found in patients who died in hospitals differed from those who died outside hospitals. Furthermore, the pre-hospital treatment of acute poisonings is substantial, and in Oslo, naloxone treatment is given by paramedics on scene [18]. Therefore, hospital statistics are not sufficiently comprehensive when discussing fatal poisonings in the context of prescription policy and drug toxicity.

Opiates or opioids were the main toxic agents in two-thirds of all fatal poisonings during the study period, and heroin or morphine accounted for the majority of these deaths. When compared with the other Nordic countries, Norway has been shown to have a higher percentage of deaths caused by heroin or morphine: 72% vs. 10% in Finland, 44% in Denmark, and 66% in Sweden in 2002 [1]. In the 2002 study, the mortality rate for fatal overdoses, as well as the absolute number of deaths, was also higher in Norway than in the other Nordic countries [1]. The high mortality rate is probably correlated to the higher percentage of opiate or opioid poisonings, because the majority of drug addicts die of drug-related causes [19,20], as also reflected by the present study.

Methadone was found in 6% of the cases in the present study, which included both substance abusers and non-abusers. However, the percentage was 15% in the study from 2002, including substance abusers from the entire country. The figures are small and would therefore be expected to vary over time. A reduction in heroin-associated deaths was followed by an increase in methadone-associated deaths in Hamburg [7] and Denmark [1]. Interestingly, the study from Hamburg found that two-thirds of all methadone deaths were drug addicts who had never been in the methadone maintenance treatment (MMT) program. However, information regarding their MMT status was not available in the present study. The relatively small percentage of methadone-associated deaths compared with the relatively high number of patients in the MMT program in Norway (4319 patients, of whom 699 were in Oslo [21], vs. 2100 in the whole of Sweden [22] in 2003), is not sufficient to dissuade MMT.

Methanol and TCAs had the highest case fatality rate during the study period. Methanol is known to be highly toxic, and in a methanol-poisoning outbreak in Norway from 2002 to 2004, a 29% mortality rate was found [23]. The methanol poisonings in the present study were part of this outbreak, and the observed 33% deaths per poisoning episode is in accordance with results for the outbreak as a whole.

Four fatalities with TCAs were found, and one with SSRIs. The emerging use of SSRIs has led to a recent controversy regarding whether these drugs increase or decrease suicide rates. However, SSRIs were suspected twice as often as TCAs in non-fatal cases. This is in accordance with previous findings suggesting that the risk of non-fatal self-harm may be increased in SSRIs [24], but TCAs are more toxic [8]. In meta-analyses of randomized controlled trials, Wayne et al. found that SSRIs might increase suicide ideation, but found no evidence that suicide risk was increased [9]. Because of a shift in prescription pattern, SSRIs are now more commonly used than older anti-depressants [5]. Therefore, it would be expected that SSRIs be found more often than TCAs in this study. However, only 26% of those who committed suicide had taken anti-depressants, supporting studies suggesting that under-treatment of depression is a greater problem than an eventual increased risk of suicide by specific compounds [25]. The present finding of anti-depressants in 25% of accidental deaths presumably reflects their therapeutic use and is possibly an indicator of depression. Furthermore, this illustrates the potential problems encountered when evaluating the intended outcome of an acute poisoning post-mortem.

Ethanol and benzodiazepines are important co-drugs in acute poisonings, but were found to be the main toxic agents in nine and four fatalities, respectively. However, because these drugs are the most commonly found in acute poisonings in Oslo [12], the percentage of deaths per poisoning episode was low, about 1%. Ethanol was the main toxic agent in 9% of all fatal poisonings and an additional agent in 17%. Enhanced respiratory depression is important in multiple-drug poisonings, both with opioids [1] and psychoactive drugs [8]. Benzodiazepines caused 4% of all fatal poisonings, in accordance with findings from England, where benzodiazepines caused 3.8% of all deaths caused by single-drug poisoning. However, 75% of all deaths had benzodiazepines as the main or additional drugs. Zopiclone is increasingly used as a sedative compared with benzodiazepines, as the potential for drug dependency is thought to be less evident. However, there were 8% deaths per poisoning episode for zopiclone vs. 1% for benzodiazepines in the present study, although others have concluded that the fatal toxicity was the same for both sedatives [26]. Acute poisoning by zopiclone mimics benzodiazepine poisoning clinically, and could have been classified as such in the non-fatal cases. Furthermore, case fatality rates were calculated for main toxic agents only, but many clinicians might have considered zopiclone a less harmful drug and therefore an additional agent in many cases, which could be a possible bias.

Paracetamol was the main agent in two fatalities but an additional agent in 11. Combinations of paracetamol and codeine were quite common. In such cases, the main agent was thought to be paracetamol in hospitalized patients, because of the potential for liver damage, and codeine in forensic cases, because of presumed respiratory depression causing death before liver failure occurred.

The proportion of hidden suicides among drug overdoses has been debated, but studies find that the majority of such events are accidental, not deliberate [2,27]. In a study by Pfab et al., patients treated for drug overdose were interviewed after awakening, and 9% reported a suicidal intention [2]. The reliability of suicide statistics in cases of acute poisonings in Norway was most recently evaluated in 1985, and the proportion of possible hidden suicides was found to be 10%, corresponding to the study by Pfab et al. [28]. Since then, the autopsy rate has declined, and only three out of 11 fatal poisonings in hospital underwent a medico-legal autopsy in this study. Therefore, due to the declining autopsy rate, the suicide rate is probably underestimated in Norway today, and the 31% classified as suicides should be considered a minimum.

Seventy per cent of the fatal cases were classified as substance abusers, mostly of illegal drugs, emphasizing the increased mortality rate among this patient group [29]. Only 10 deaths were classified as non-abusers, of which nine were evaluated as suicides. Of course, no information regarding previous substance abuse would make it more unlikely to consider the cause of death accidental and vice versa. However, it is worth emphasizing that 34% of those who committed suicide were classified as abusers. This is in accordance with other studies indicating substance abuse as the second most common precursor to suicide [30].

### Strengths and limitations

The major strength of this study was the inclusion of all acute poisonings in Oslo during one year. The inclusion of all acute poisonings, both fatal and non-fatal, within a defined geographical area made it possible to generalize to the general population of the city of Oslo, thus minimizing selection bias. The completeness of the inclusion of patients in these types of studies can always be questioned. However, we included patients at three levels of healthcare in this multi-centre study, and transferrals between these levels were common. This helped to make the study more complete because each patient could have been included in up to three treatment facilities during each episode. Each poisoning episode was traced through the system, thus making comparison between fatal and non-fatal poisonings possible. We believe the numbers to be as close to reality as possible, although there is still a possibility that some cases might have been missed.

The major limitation was that extensive laboratory testing to identify the toxic agents was conducted only for the cases undergoing a medico-legal autopsy. Blood or urine screening has only a limited value in the treatment of acute poisonings as long as the treatment is mainly symptomatic and guided by clinical signs or symptoms. Therefore, the classification of main or additional agents in non-fatal poisonings was based on a clinical evaluation and some laboratory analyses, if appropriate. However, for the hospitalized patients, a serum laboratory test for the eight most common agents was performed, and this showed a good sensitivity for the most common agents [31]. Unlike the deceased, hospitalized patients could give information about drug intake, and the need for extensive testing was therefore not necessary for the diagnosis of acute poisoning. Apart from serum ethanol concentrations, serum paracetamol concentrations and other toxic agents analysed on request (such as lithium), laboratory tests would not give a reliable immediate answer. However, results from arterial blood gas and other clinical parameters were available. The treatment of acute poisonings is based on the clinical evaluation of the patient, combined with routine laboratory testing [32]. Most drugs identified in studies using more extensive laboratory testing were additional drugs, and finding them would not have altered treatment [33-35]. We therefore chose this clinical definition. As for the evaluation of intention, no form or scale was used. This is how the fatal and the non-fatal poisonings are evaluated in clinical practice, and therefore a generalization about the general population could be made. For case-fatality rates, only main toxic agents were used. Some toxic agents, such as benzodiazepines, tend to be classified as additional agents in non-fatal cases, and this might be a possible bias for the calculated case-fatality rates.

Further studies are needed to address the relative importance of different toxic agents, especially related to the availability of the drugs. Prescription pattern, preferably for each patient, would add important information regarding anti-depressant treatment and whether the opioids or benzodiazepines used were prescribed medication or acquired illegally.

## Conclusions

Acute poisonings were fatal in 3% of the registered poisoning episodes in Oslo during one year, and victims were either treated by the health care system or found dead outside hospital. The majority died of opiate or opioid poisoning, and seven out of 10 deceased were classified as drug dependent. One-third were evaluated as suicides. The great majority of deaths occurred outside hospitals. When compared with non-fatal poisonings, methanol and TCAs were most toxic, i.e. had the highest percentage of fatal cases. Both fatal and non-fatal poisonings need to be included when discussing toxicity and drug use patterns.

## Competing interests

The authors declare that they have no competing interests.

## Authors' contributions

MAB structured the data, performed the statistical analyses, and drafted the manuscript. BT collected the data for the fatal poisonings. KEH participated in the planning of the study and co-ordinated the collection of data. FH structured the data files on non-fatal poisonings. OE participated in the design of the study and supervised the work. DJ conceived the study and supervised the work. All authors participated in drafting the manuscript and read and approved the final version.

## Pre-publication history

The pre-publication history for this paper can be accessed here:

http://www.biomedcentral.com/1471-227X/10/13/prepub

## References

[B1] Steentoft A Teige B Holmgren P Vuori E Kristinsson J Hansen AC Ceder G Wethe G Rollmann D Fatal poisoning in Nordic drug addicts in 2002 Forensic Sci Int 2006 160 148 156 10.1016/j.forsciint.2005.09.004 16289615

[B2] Pfab R Eyer F Jetzinger E Zilker T Cause and motivation in cases of non-fatal drug overdoses in opiate addicts Clin Toxicol (Phila) 2006 44 255 259 16749542 10.1080/15563650600584394

[B3] Retterstol N Ekeberg O Mehlum L Selvmord - et personlig og samfunnsmessig problem 2002 Oslo, Gyldendal Akademisk

[B4] Heyerdahl F Bjornaas MA Dahl R Hovda KE Nore AK Ekeberg O Jacobsen D Repetition of acute poisoning in Oslo: 1-year prospective study Br J Psychiatry 2009 194 73 79 10.1192/bjp.bp.107.048322 19118331

[B5] Nordentoft M Qin P Helweg-Larsen K Juel K Time-trends in method-specific suicide rates compared with the availability of specific compounds. The Danish experience Nord J Psychiatry 2006 60 97 106 10.1080/08039480600600169 16635927

[B6] Baumert J Erazo N Ruf E Ladwig KH Time trends in suicide mortality vary in choice of methods: an analysis of 145,865 fatal suicide cases in Germany 1991-2002 Soc Psychiatry Psychiatr Epidemiol 2008 43 913 919 10.1007/s00127-008-0380-7 18560783

[B7] Heinemann A Iwersen-Bergmann S Stein S Schmoldt A Puschel K Methadone-related fatalities in Hamburg 1990-1999: implications for quality standards in maintenance treatment? Forensic Sci Int 2000 113 449 455 10.1016/S0379-0738(00)00282-6 10978661

[B8] Flanagan RJ Fatal toxicity of drugs used in psychiatry Hum Psychopharmacol 2008 23 Suppl 1 43 51 10.1002/hup.916 18098225

[B9] Hall WD Lucke J How have the selective serotonin reuptake inhibitor antidepressants affected suicide mortality? Aust N Z J Psychiatry 2006 40 941 950 17054562 10.1080/j.1440-1614.2006.01917.x

[B10] Lahti RA Vuori E Fatal drug poisonings: medico-legal reports and mortality statistics Forensic Sci Int 2003 136 35 46 10.1016/S0379-0738(03)00223-8 12969618

[B11] Lilleeng GH Berg KJ Gjertsen F Andrew E [Acute poisonings in Norway 1999-2004--morbidity and mortality] Tidsskr Nor Laegeforen 2007 127 1023 1027 17457385

[B12] Hovda KE Bjornaas MA Skog K Opdahl A Drottning P Ekeberg O Jacbsen D Acute poisonings treated in hospitals in Oslo: a one-year prospective study (I): pattern of poisoning Clin Toxicol (Phila) 2008 46 35 41 18167035 10.1080/15563650601185969

[B13] Heyerdahl F Hovda KE Bjornaas MA Nore AK Figueiredo JC Ekeberg O Jacobsen D Pre-hospital treatment of acute poisonings in Oslo BMC Emerg Med 2008 8 15 10.1186/1471-227X-8-15 19025643 PMC2605443

[B14] World Health Organisation International Classification of Diseases - version 10 1996 annual update 2008

[B15] Rygnestad T A prospective 5-year follow-up study of self-poisoned patients Acta Psychiatrica Scandinavica 1988 77 328 331 10.1111/j.1600-0447.1988.tb05129.x 3394534

[B16] Burillo-Putze G Munne P Duenas A Pinillos MA Naveiro JM Cobo J Alonso J National multicentre study of acute intoxication in emergency departments of Spain Eur J Emerg Med 2003 10 101 104 10.1097/00063110-200306000-00006 12789064

[B17] Heyerdahl F Bjornas MA Hovda KE Skog K Opdahl A Wium C Ekeberg O Jacobsen D Acute poisonings treated in hospitals in Oslo: a one-year prospective study (II): clinical outcome Clin Toxicol (Phila) 2008 46 42 49 18167036 10.1080/15563650701210048

[B18] Buajordet I Naess AC Jacobsen D Brors O Adverse events after naloxone treatment of episodes of suspected acute opioid overdose Eur J Emerg Med 2004 11 19 23 10.1097/00063110-200402000-00004 15167188

[B19] Ghodse H Oyefeso A Kilpatrick B Mortality of drug addicts in the United Kingdom 1967-1993 Int J Epidemiol 1998 27 473 478 10.1093/ije/27.3.473 9698138

[B20] Bargagli AM Hickman M Davoli M Perucci CA Schifano P Buster M Brugal T Vicente J Drug-related mortality and its impact on adult mortality in eight European countries Eur J Public Health 2006 16 198 202 10.1093/eurpub/cki168 16157612

[B21] SIRUS Norwegian Institute for Alcohol and Durg Research: Alcohol and Drugs in Norway 2004 Annual Report. Oslo 2004

[B22] European Monitoring Centre for Drugs and Drug addiction Drug treatment overviews: Sweden Annual report. Lisbon 2009

[B23] Hovda KE Hunderi OH Tafjord AB Dunlop O Rudberg N Jacobsen D Methanol outbreak in Norway 2002-2004: epidemiology, clinical features and prognostic signs J Intern Med 2005 258 181 190 10.1111/j.1365-2796.2005.01521.x 16018795

[B24] Martinez C Rietbrock S Wise L Ashby D Chick J Moseley J Evans S Gunnell D Antidepressant treatment and the risk of fatal and non-fatal self harm in first episode depression: nested case-control study BMJ 2005 330 389 10.1136/bmj.330.7488.389 15718538 PMC549107

[B25] Isacsson G Holmgren P Ahlner J Selective serotonin reuptake inhibitor antidepressants and the risk of suicide: a controlled forensic database study of 14,857 suicides Acta Psychiatr Scand 2005 111 286 290 10.1111/j.1600-0447.2004.00504.x 15740464

[B26] Reith DM Fountain J McDowell R Tilyard M Comparison of the fatal toxicity index of zopiclone with benzodiazepines J Toxicol Clin Toxicol 2003 41 975 980 10.1081/CLT-120026520 14705844

[B27] Kjelsberg E Winther M Dahl AA Overdose deaths in young substance abusers: accidents or hidden suicides? Acta Psychiatr Scand 1995 91 236 242 10.1111/j.1600-0447.1995.tb09775.x 7625204

[B28] Ekeberg O Jacobsen D Enger E Frederichsen P Holan L [The reliability of suicide statistics in Norway] Tidsskr Nor Laegeforen 1985 105 123 127 3975879

[B29] Bjornaas MA Bekken AS Ojlert A Haldorsen T Jacobsen D Rostrup M Ekeberg O A 20-year prospective study of mortality and causes of death among hospitalized opioid addicts in Oslo BMC Psychiatry 2008 8 8 10.1186/1471-244X-8-8 18271956 PMC2277385

[B30] Murphy GE Hawton K, Van Heeringen K Psychiatrics aspects of suicidal behaviour: Substance abuse The International Handbook of Suicide and Attempted Suicide 2000 West Sussex: John Wiley & Sons Ltd 136 146

[B31] Heyerdahl F Hovda KE Bjornaas MA Brors O Ekeberg O Jacobsen D Clinical assessment compared to laboratory screening in acutely poisoned patients Hum Exp Toxicol 2008 27 73 79 10.1177/0960327107087800 18480152

[B32] Rygnestad T Aarstad K Gustafsson K Jenssen U The clinical value of drug analyses in deliberate self-poisoning Hum Exp Toxicol 1990 9 221 230 10.1177/096032719000900404 2390319

[B33] Pohjola-Sintonen S Kivisto KT Vuori E Lapatto-Reiniluoto O Tiula E Neuvonen PJ Identification of drugs ingested in acute poisoning: correlation of patient history with drug analyses Ther Drug Monit 2000 22 749 752 10.1097/00007691-200012000-00016 11128245

[B34] Skelton H Dann LM Ong RT Hamilton T Ilett KF Drug screening of patients who deliberately harm themselves admitted to the emergency department Ther Drug Monit 1998 20 98 103 10.1097/00007691-199802000-00018 9485563

[B35] Brett AS Implications of discordance between clinical impression and toxicology analysis in drug overdose Arch Intern Med 1988 148 437 441 10.1001/archinte.148.2.437 3341840

